# National Association of Dental Examiners

**Published:** 1905-04

**Authors:** Chas. C. Chittenden

**Affiliations:** Chairman Committee on Colleges


### Domestic Correspondence.

### NATIONAL ASSOCIATION OF DENTAL EXAMINERS.

To the Editor:

Sir,—In consideration of the conflicting views as to dental educational standards which have existed for some time, the National Association of Dental Examiners, at its annual meeting held at St. Louis, August, 1904, deemed it expedient, and necessary for the upholding of such schools as sought to maintain the standards already published to the world as the minimum that should obtain, to declare what educational standards should be required by the State Boards of Examiners as a criterion of reputability of the schools seeking recognition of their output.

This *ad interim* committee, which is also the Committee on Colleges, was instructed to inform all schools of the action taken, and directed to prepare a recommended list of colleges on the basis of the standards established at that meeting.

Feeling fully the gravity of the duty imposed, this committee has expended much effort in striving to arrive at a basis of fairness to all interests concerned in carrying out its general instructions. The chief requirement established at St. Louis was that of "graduation from an accredited high school or its full equivalent" for admission to the classes of 1905–06.

In several schools and university departments this requirement is already in actual operation, and our committee finds a considerable number of other schools desiring to maintain it. All these, of course, will be placed on the recommended list. There are, however, other schools whose deans assert that to enforce at once this advance requirement would work a serious financial injury to their institutions.

The question of what should constitute a proper length of course for graduation from dental colleges has always been left by the examiners to the colleges themselves, except that, after a school has announced to the public a certain course as necessary to properly fit a student for graduation, if it for private or financial reasons deliberately lowers its requirements in any particular, the question of good faith and reputability of that school becomes at once a matter for adjudication by every board in the country.

We, therefore, acting upon authority of, and *ad interim* representing the National Association of Dental Examiners, which is the advisory body of the various State boards in their official acts, respectfully request that you authorize the Committee on Colleges to place your school on the recommended list of colleges by the acceptance of the following educational requirements for students, —viz.:

For matriculation or registration, "Graduation from an accredited high school or its full equivalent, all examination of credentials and equivalents to be placed in the hands of an acceptable appointee of the State Superintendent of Public Instruction where not otherwise provided for by law," said requirements to be inaugurated not later than the beginning of the school year of *1906–07;* and a college course for graduation optional with you of either four years of seven months each or three years of nine months each, this course requirement to be inaugurated the present year, *1905.*

It is to be expected that schools maintaining these standards will be protected in so doing by the several boards composing the National Association of Dental Examiners.

It is the intention of this Committee to prepare and publish the recommended list of colleges not later than April 1 next, in order to give all schools the earliest opportunity to announce these standards to the public. Therefore information as to your decision is desired as early as possible.

Very respectfully yours,

CHAS. C. CHITTENDEN,
*Chairman Committee on Colleges.*

